# Prevalence of germline pathogenic variants in 22 cancer susceptibility genes in Swedish pediatric cancer patients

**DOI:** 10.1038/s41598-021-84502-4

**Published:** 2021-03-05

**Authors:** Kristoffer von Stedingk, Karl-Johan Stjernfelt, Anders Kvist, Cecilia Wahlström, Ulf Kristoffersson, Marie Stenmark-Askmalm, Thomas Wiebe, Lars Hjorth, Jan Koster, Håkan Olsson, Ingrid Øra

**Affiliations:** 1 Department of Pediatrics, Clinical Sciences, Lund University, Lasarettsgatan 40, 22185 Lund, Sweden; 2 Pediatric Oncology and Hematology, Children’s Hospital, Skåne University Hospital, Lund, Sweden; 3 Department of Oncogenomics, University Medical Center, AMC, University of Amsterdam, Amsterdam, The Netherlands; 4 Department of Oncology and Pathology, Clinical Sciences, Lund University, Lund, Sweden; 5 Department of Laboratory Medicine, Division of Clinical Genetics, Lund University, Lund, Sweden; 6 Department of Oncology, Clinical Sciences, Lund University, Lund, Sweden; 7 Department of Cancer Epidemiology, Clinical Sciences, Lund, University, Lund, Sweden

**Keywords:** Cancer genetics, Cancer screening, Cancer, Paediatric cancer

## Abstract

Up to 10% of pediatric cancer patients harbor pathogenic germline variants in one or more cancer susceptibility genes. A recent study from the US reported pathogenic variants in 22 out of 60 analyzed autosomal dominant cancer susceptibility genes, implicating 8.5% of pediatric cancer patients. Here we aimed to assess the prevalence of germline pathogenic variants in these 22 genes in a population-based Swedish cohort and to compare the results to those described in other populations. We found pathogenic variants in 10 of the 22 genes covering 3.8% of these patients. The prevalence of *TP53* mutations was significantly lower than described in previous studies, which can largely be attributed to differences in tumor diagnosis distributions across the three cohorts. Matched family history for relatives allowed assessment of familial cancer incidence, however, no significant difference in cancer incidence was found in families of children carrying pathogenic variants compared to those who did not.

## Introduction

Pediatric cancer is linked to a number of inherited disorders including Li-Fraumeni syndrome, retinoblastoma and neurofibromatosis. However, these and other germline predisposition syndromes explain a small proportion of pediatric cancers, currently estimated to account for 10% of cases^1,2^.

In 2008 we initiated the Lund Childhood Cancer Genetics (LCCG) study, the aim of which was to prospectively include all pediatric patients diagnosed with cancer in southern Sweden^3,4^. We have reported that approximately 5% of pediatric cancer patients in this population-based cohort have a pediatric relative with the same disease diagnosis within third-degree relations, most often found among patients with leukemia and CNS tumors. Furthermore, we observed a significant female predominance among familial pediatric leukemia and CNS cancer patients in families with more than one pediatric cancer case^4^.

In a study carried out by Zhang et al. in 2015, blood samples were collected from a cohort of 1120 pediatric and young adult cancer patients from the US and examined using WGS and/or exome sequencing. They identified pathogenic or likely pathogenic variants in 8.5% of their cohort^5^. The reported variants were detected in 21 of the 60 autosomal dominant cancer predisposition genes analyzed, the most frequently affected of which was *TP53*. A biallelic pathogenic variant was also found in *ATM*, although this gene was not investigated as an autosomal dominant cancer predisposition gene. Among the patients presenting with germline pathogenic mutations in cancer-associated genes, only 40% had a reported family history of cancer, which is not significantly higher than in those patients with no identifiable germline mutations. In another recent comprehensive analysis of 914 children and young adult cancer patients compiled from various sources, the majority of which were German, Gröbner et al. reported that approximately 6% of patients harbored a cancer predisposing germline variant^6^.

In the present study, we performed targeted sequencing of the 22 genes with pathogenic and likely pathogenic variants reported by Zhang et al.^5^ in 790 blood samples from the LCCG cohort of pediatric cancer patients. The aim was to estimate the prevalence of germline pathogenic variants in these genes in a population-based Swedish cohort. By doing so, we aim to compare the results to those in the studies by Zhang et al. and Gröbner et al., as well as elucidate potential differences in the prevalence of mutations in these predisposition genes in different populations.

## Results

### Patient cohort

**Figure 1:**
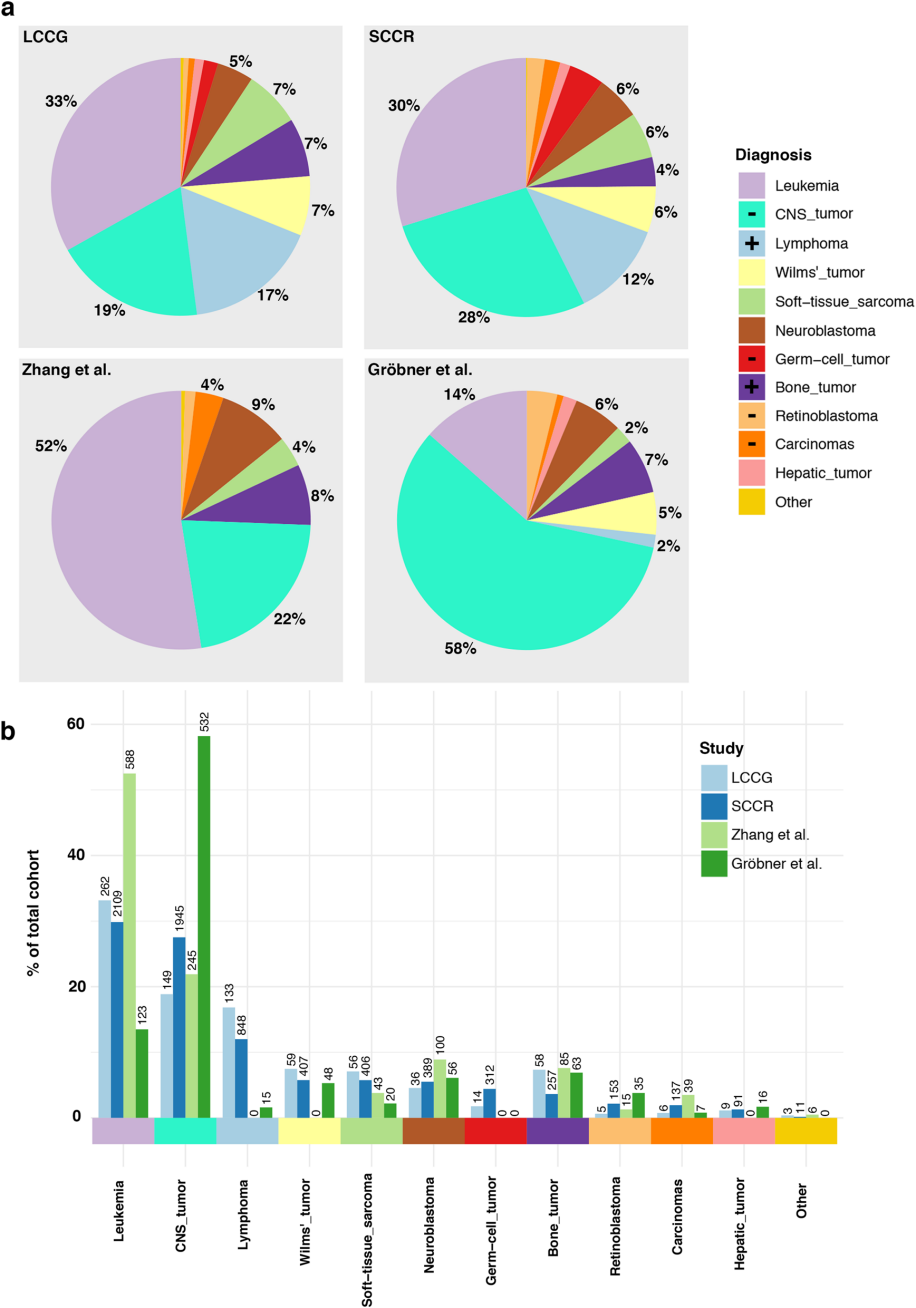
Pediatric cancer diagnosis distributions. (**a**) Distributions of the current LCCG cohort (n = 790; top left), the SCCR cohort (n = 7065; top right), the Zhang cohort (n = 1120; bottom left) and the Gröbner cohort (n = 914; bottom right). + and − indicate significant over- or under-representation of diagnoses in the LCCG cohort compared to the Swedish Childhood Cancer Registry (SCCR) cohort. Diagnosis percentage of each cohort for the largest diagnoses are displayed on each respective pie chart. (**b**) Distribution bar-plot of all cohorts divided according to diagnosis. Number of patients in each cohort diagnosis group is displayed above each bar. Comparative statistics (Fisher’s Exact test) are provided in Supplementary Table S1.

Our study includes 790 pediatric cancer patients from the LCCG study^7^ (referred to as the LCCG cohort). All were under the age of 18 years at diagnosis and the most prevalent cancers are leukemia and CNS tumors (33% and 19%, respectively) (Fig. 1, Table 2). Compared to the distribution of pediatric cancers in the general Swedish population (according to the Swedish Childhood Cancer Registry (SCCR) 2013 Report), the LCCG cohort contains lower proportions of CNS tumors (19% vs. 28%), germ-cell tumors, retinoblastoma and carcinomas, and higher proportions of lymphomas (17% vs. 12%), and bone tumors (Fig. 1, Supplementary Table S1). Greater differences were observed when comparing the distribution of diagnoses in our cohort to the two recent childhood cancer studies published by Zhang et al. in 2015 (referred to as the Zhang cohort) and Gröbner et al. in 2018 (referred to as the Gröbner cohort)^5,6^ (Fig. 1, Supplementary Table S1). The Zhang cohort of US patients has a higher proportion of leukemia patients (53%), as well as a higher percentage of adrenocortical tumors (ACT, 3.5%), than in the LCCG cohort^8^. In Sweden, ACT accounts for a mere 1–2% of already rare childhood carcinomas, and the LCCG cohort contains only 1 ACT patient. (These differences will be discussed below in the context of the frequency of *TP53* mutations.) The study by Gröbner et al., which includes samples from multiple centers across Europe and the US, has a large proportion of CNS tumors (58%) and a low percentage of leukemia cases (13.5%). It should also be noted that both the Zhang and the Gröbner cohort contained a small proportion of young adults (Zhang; up 20 years of age, Gröbner; up to 25 years of age), while our LCCG cohort consisted exclusively of patients under 18 years of age at diagnosis.

### Target enrichment and sequencing

At least two replicate sequencing libraries were prepared and sequenced for each of the 797 DNA samples (Supplementary Table S2). All samples passed our minimum base quality score requirement of 80% of bases of base quality 30 or higher. However, less than 90% of the assay target region was covered by 30 high-quality aligned reads in seven samples. These seven samples were therefore excluded from further analyses. In the remaining 790 samples, 94.6% of the target region was covered by 30 or more high-quality aligned reads, on average, and the mean sequence coverage was 1741 reads. Only 1.1% of the assay target region had no coverage, on average per sample (Supplementary Table S7).

### Spectrum of genetic variation and detected variants

We identified 1429 genetic variants in the 22 targeted genes (Table 1 & Supplementary Tables S8 and S10). Of these, 416 were common variants (allele frequency (AF) in the Genome Aggregation Database (gnomAD ≥ 1%)), 563 were uncommon (AF < 1% and AF ≥ 0.01%), and 450 were rare (AF < 0.01%). Most of the variants in the coding region were missense (372; 72 common, 166 uncommon and 134 rare), but we also identified 9 frameshift deletions, 10 stop-gain variants, and 3 in-frame deletions. Another 53 variants were found within the splice region of an intron (the first 8 bases or the last 17 bases of an intron), and 6 of these affected the two canonical splice donor and acceptor bases adjacent to the exon border. The remaining variants were identified in UTR regions, in introns, or up- or downstream of the target genes (Supplementary Table S9).

**Table 1:** List of sequenced autosomal dominant predisposition genes.

ALK	CDH1	NF2	RB1	TP53
APC	KRAS	NRAS	RET	VHL
ATM^A^	MSH2	PALB2	RUNX1	
BRCA1	MSH6	PMS2	SDHA	
BRCA2	NF1	PTCH1	SDHB	

^A^*ATM* found to be biallelic pathogenic variant by Zhang et al.

Each individual carried, on average, 118 variant alleles in the targeted region, of which 96% were common (AF > 1%) and 2.2% were rare (AF < 0.01%). About one fifth of the individuals (168) carried one or more private variants not found in any other individual in this study or in gnomAD. Averaged over the assay target region covering 111 kilobases, the rate of variation was 1.00 per kilobase for single nucleotide variants (SNVs), and 0.07 per kilobase for non-SNVs, on average, per individual (Supplementary Table S9).

A clear majority of all variants were classified as benign or likely benign (73.8%). Pathogenic and likely pathogenic variants comprised 9 stop-gain variants, 6 frameshift variants, 5 missense variants and 5 variants affecting splicing (Supplementary Figure S3). Of these 25 pathogenic and likely pathogenic variants, 23 were rare variants (AF < 0.0001) and 2 were uncommon (AF AF < 1% and AF ≥ 0.01%) (Supplementary Tables  S3 and S10). The two uncommon ones affect SDHA and PMS2-both genes in which pathogenic variants have low to moderate penetrance for their respective associated diagnoses in heterozygous carriers and pathogenic variants can have relatively high population prevalence^9–11^. These 25 variants were detected in a total of 30 patients, indicating that germline pathogenic variants were present in 3.8% of childhood cancers in the LCCG cohort (Supplementary Table S3). The remaining 349 variants (24.4%) were classified as being of uncertain significance (Supplementary Table S8). Among rare variants, 223 were classified as (likely) benign, 204 as of uncertain significance and 23 as pathogenic or likely pathogenic. The other two pathogenic variants were categorized as uncommon (Supplementary Table S8).

**Figure 2:**
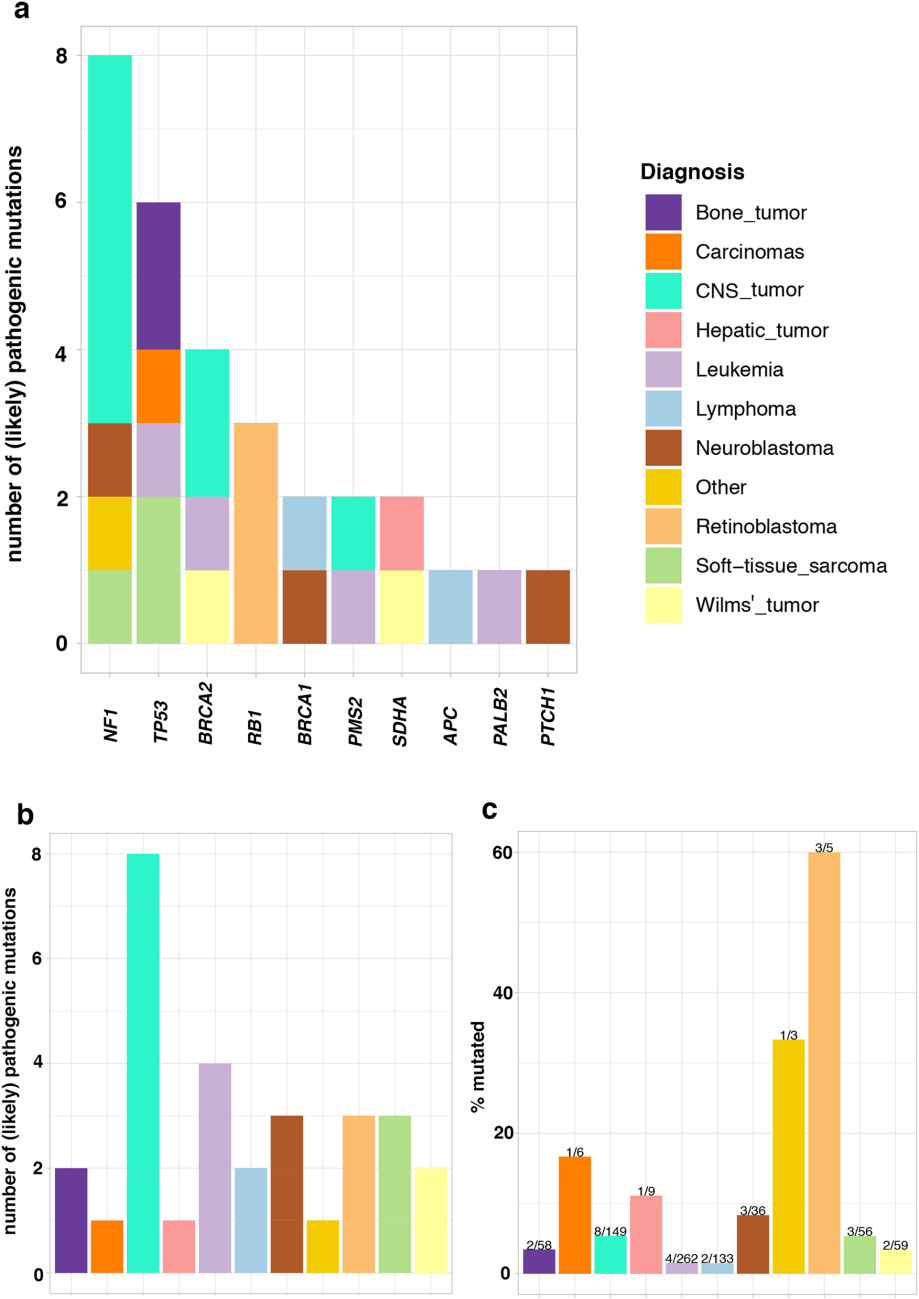
Distribution of germline pathogenic and likely pathogenic variants in patients with different pediatric diagnoses in the LCCG cohort. (**a**) Number of patients with (likely) pathogenic variants per gene. Colors indicate the diagnosis group of each patient in which the variant was detected. (**b**) Total number of patients carrying (likely) pathogenic variants per cancer diagnosis group for all genes summed. (**c**) Percentage of patients with (likely) pathogenic variant per cancer diagnosis group for all genes summed. The number of patients carrying (likely) pathogenic variants and the total number of patients in each diagnosis group is shown above the bars.

Pathogenic or likely pathogenic variants were detected in 10 of the 22 analyzed genes (Fig. 2, Table 2, Supplementary Table S3). *NF1* showed the highest prevalence (n = 8; 1% of cases); the majority of patients having CNS tumors. *TP53* had the second highest prevalence (n = 6; 0.76%), with patients showing a variety of different tumor types, including soft-tissue sarcomas, osteosarcoma, leukemia, and carcinoma (in this case an ACT). *BRCA2* pathogenic variants were found in 4 patients (0.5%), with diagnoses including CNS tumors, Wilms’ tumor and leukemia. Pathogenic variants in *RB1* were detected in 3 patients, followed by *SDHA*, *BRCA1,* and *PMS2* each with 2 patients, while individual patients exhibited pathogenic or likely pathogenic variants in *APC*, *PALB2*, and *PTCH1*.

**Table 2:** Diagnosis distribution of LCCG cohort including subgroups with corresponding germline mutations**.**

Group #	Diagnosis	n^A^	Diagnosis Subgroup	n^B^	nMut^C^	Germline Mutations
1	Leukemia	262	ALL	219	2	*BRCA2, TP53*
			AML	37	2	*PMS2, PALB2*
			Other	6	0	na
2	CNS_tumor	149	Astrocytoma	61	4	2 × *BRCA2, PMS2, NF1*
			Ependyoma	14	0	na
			Medulloblastoma	25	0	na
			Ganglioma	6	0	na
			Craniopharyngioma	6	0	na
			Opticusglioma	9	3	3 × *NF1*
			Other_Brain_tumor	28	1	*NF1*
3 & 9	Lymphoma	133	NHL	56	2	*APC, BRCA1*
			Hodgkin's_lymphoma	58	0	na
			Histiocytosis	19	0	na
4	Wilms'_tumor	59	Wilms'_tumor	59	2	*BRCA2, SDHA*
5	Soft-tissue_Sarcoma	56	Non-rhabdomyosarcoma	16	1	TP53
			Rhabdomyosarcoma_Embryonal	30	1	TP53
			Rhabdomyosarcoma_Alveolar	7	0	na
			Rhabdomyosarcoma_Unspecified	3	1	*NF1*
6	Neuroblastoma	36	Neuroblastoma	36	3	*BRCA1, PTCH1, NF1*
7	Germ-cell_tumor	14	Germ-cell_tumor	14	0	na
8	Bone_tumor	58	Osteosarcoma	22	2	2 × *TP53*
			Ewing_sarcoma	36	0	na
10	Retinoblastoma	5	Retinoblastoma	5	3	3 × *RB1*
11	Carcinoma	6	ACT	1	1	*TP53*
			Other Carcinoma	5	0	*na*
12	Hepatic_tumor	9	Hepatoblastoma	9	1	*SDHA*
13	Other	3	Other	3	1	*NF1*

*ALL* acute lymphoblastic leukemia, *AML* acute myeloid leukemia, *NHL* non-Hodgkin's lymphoma.

^A^Number of patients within each main diagnosis group.

^B^Number of patients in each diagnosis subgroup.

^C^Number of (likely) pathogenic mutations.

### Prevalence of pathogenic variants by tumor type

Retinoblastoma patients exhibited the highest prevalence of germline pathogenic or likely pathogenic variants; three out of five patients (60%) carrying variants in the disease-associated *RB1* gene (Fig. 2)^12^. Three out of 36 neuroblastoma patients (8%) carried germline pathogenic variants in *NF1, BRCA1*, and *PTCH1*. Germline pathogenic variants in *NF1* have been described previously in neuroblastoma patients^13,14^. Among 56 patients with soft-tissue sarcoma, we found three carrying pathogenic or likely pathogenic variants (5%), all in genes previously linked to this tumor type (*TP53*: 2 patients; *NF1*: 1 patient)^15–17^. Patients with CNS tumors harbored germline pathogenic or likely pathogenic variants in 8 of 149 cases (5%): five in *NF1*, two in *BRCA2* and one in *PMS2*; all genes previously reported in patients with CNS tumors^5,18–20^. Four of 268 patients (1.5%) with leukemia carried pathogenic or likely pathogenic variants in *TP53*, *BRCA2*, *PALB2,* and *PMS2*. Only *TP53* is associated with susceptibility to leukemia^21^, although pathogenic variants in *BRCA2* and *PALB2* have been reported previously in leukemia patients*.* Two of the 59 patients (3%) with Wilms’ tumor carried pathogenic variants in *BRCA2* and *SDHA*.

Two patients carrying pathogenic variants in *TP53* were found among 58 patients (3.4%) with bone tumors, which are associated with Li-Fraumeni syndrome^5,22^. Single patients with pathogenic variants in *TP53* and a likely pathogenic variant in *SDHA* were found among the seven carcinomas and nine hepatic tumors, respectively. The *TP53* variant was found in an ACT case, a tumor type associated with germline *TP53* pathogenic variants^5,17,20^.

### Comparison with previous studies

In order to more accurately compare cohorts, we examined only variants that were screened for in all three studies. As our screening methods do not detect copy number variations (CNVs), CNV variants from the Zhang et al. and Gröbner et al. cohorts were excluded. We found a lower overall prevalence of pathogenic and likely pathogenic variants in the 22 screened genes in our LCCG cohort than in the US-based study by Zhang et al. (OR 2.2, FDR-adjusted *p*-value = 0.001) and in the multi-center study by Gröbner et al. (OR = 1.8, FDR-adjusted *p*-value = 0.028) (Supplementary Table S5. No significant difference was detected between the Zhang and Gröbner cohorts.

**Figure 3:**
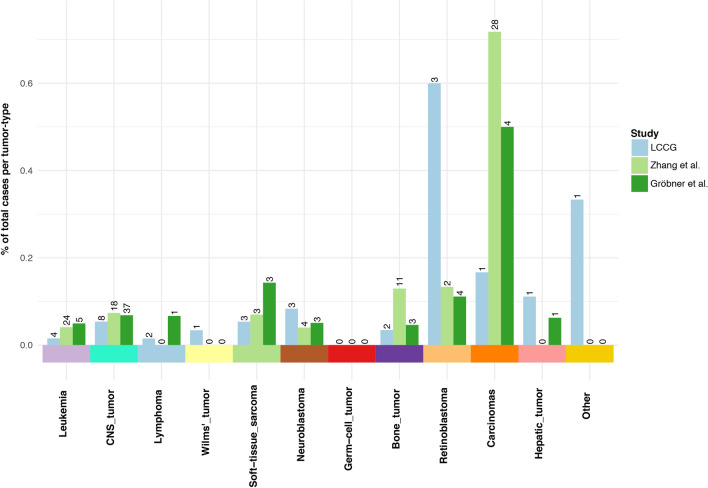
Mutation prevalence per tumor-type. Distribution bar-plot of LCCG, Zhang et al. and Gröbner et al. cohorts divided according to diagnosis. Number of (likely) pathogenic variants in each cohort diagnosis group is displayed above each bar. Comparative statistics are provided in Supplementary Table S6.

As shown above, the distribution of diagnoses differs substantially between the cohorts and this could influence both the distribution and prevalence of pathogenic variants in the analyzed genes. Examining the overall prevalence of pathogenic variants within each diagnosis subgroup we find no significant differences across the three cohorts (Fig. 3, Supplementary Table S6).

On an individual gene basis, the only difference between the LCCG cohort and the Zhang and Gröbner cohorts was the prevalence of *TP53* mutations, although this difference with the Gröbner cohort was not significant after FDR-adjustment of the *p*-values (LCCG cohort vs. Zhang cohort: OR = 5.8, FDR-adjusted *p*-value < 0.001; LCCG cohort vs. Gröbner cohort: OR = 3.5, FDR-adjusted *p*-value = 0.154). Again, no significant differences were found between the Zhang and Gröbner cohorts for any of the genes (Supplementary Table S5). Exclusion of *TP53* from the comparisons removed any statistical differences in aggregate prevalence for all the genes between the studies. It should be noted that the number of carriers of mutations in all genes other than *TP53* were low, and a much larger cohort size would be required to identify any true underlying differences in prevalence between the populations.

While the lower prevalence of *TP53* mutations in our study could be attributed to a true lower population burden, it could also be due to differences in mutation classification across studies. To determine whether such differences in criteria for classification of variant pathogenicity contributed to the observed differences in prevalence between the LCCG cohort and the Zhang cohort, we re-classified all pathogenic *TP53* mutations reported by Zhang et al. (information required for re-classification was not available for the Gröbner cohort). Six of the 22 missense variants reported by Zhang et al. as pathogenic were classified as being of uncertain significance according to our criteria, reducing the number of *TP53* carriers from 48 to 42. However, this only explained a small proportion of the difference, and a significant difference in the prevalence of the *TP53* mutation remained between our cohort and that of Zhang et al. (*P* < 0.0001, OR = 5.08).

Both Zhang et al. and Gröbner et al. observed the highest prevalence of *TP53* mutations (69% and 50%, respectively) in ACTs, which accounted for 3.5% (n = 39) and 0.9% (n = 8) of their cohorts, respectively. Only one ACT was found in our cohort. Excluding ACTs from the Zhang et al. and Gröbner et al. cohorts removed the difference in the prevalence of *TP53* mutations in both studies after FDR-adjustment of the *p*-values (Supplementary Table S5).

### Family history of cancer

In our total cohort of 790 patients, data on family history of cancer were available for 86% of the patients (n = 676/790). Overall, 28% (n = 190/676) of patients had a first-degree relative with a cancer diagnosis, and 83% (n = 560/676) had a cancer diagnosis in the family up to the second-degree of relation. We further divided the cohort into those with and without mutations in the examined cancer susceptibility genes. In patients with detected mutations, family history data were available for 80% (n = 24/30), of which 46% (n = 11) had a family history of cancer within first-degree relatives, and 96% (n = 23/24) within second-degree relatives. In patients without detected cancer susceptibility gene mutations, family history data were available for 86% (n = 652/760), of which only 27% (n = 179/652) had a first-degree relative with cancer and 82% (n = 537/652) within the second-degree. Neither the observed differences in first-degree relatives with cancer diagnoses nor second-degree relatives were significantly higher in patients with a detected mutation (Fisher’s exact *P* = 0.06, OR = 2.23, and *P* = 0.10, OR = 4.92, respectively). This observation is also in line with the findings of Zhang et al. who reported no difference. It is notable that in the US study, Zhang et al. found a family history of cancer within the first-degree in 42% of patients without germline mutations, which is higher than the 27% observed in our cohort (*P* = 0.054, OR = 0.53). No significant difference was observed in the prevalence of germline mutations between genders (Fisher’s exact *P* = 0.71, OR = 0.86).

## Discussion

We have performed targeted DNA sequencing of 22 previously described autosomal dominant cancer predisposition genes^5^ in blood samples collected from 790 pediatric cancer patients diagnosed in southern Sweden. We found that 3.8% of patients in this cohort harbored germline pathogenic or likely pathogenic variants in one of the 22 cancer predisposition genes examined. This is lower than that reported in two recent studies of pediatric and young adult cancer patients, where pathogenic or likely pathogenic variants in these 22 genes were found in 6.7% (Zhang cohort) and 8.0% (Gröbner cohort) of cases (excluding 5 copy number variants because this type of variant is not detectable with our assay)^5,6^. On an individual gene basis, the only significant difference between the three cohorts was the prevalence of *TP53* mutations, and removing this gene from the comparison removed the significant difference in the aggregate prevalence of pathogenic variants between the cohorts. Both the Gröbner and the Zhang cohorts had a substantially higher proportion of ACTs than our cohort, which in both cases was associated with the highest rates of *TP53* mutations, ranging from 50 to 69%, respectively. Zhang et al. acknowledged the fact that their cohort included a greater-than-expected proportion of patients with ACTs and hypodiploid acute lymphoblastic leukemia. When these were excluded, the germline mutation rate was 5.6%, which is comparable to that in the study by Gröbner et al. Our cohort contained only one case of ACT, which, as may be expected, did indeed harbor a germline *TP53* mutation. Adjusting for discrepancies in ACT patients across all studies showed that it was a significant contributing factor to the discrepancy in *TP53* mutations across the three studies.

The comparison between our cohort and that of Zhang et al. is inherently biased because we chose to screen only the 22 genes in which Zhang et al. had found pathogenic variants, causing a regression towards the mean type of bias. The comparison is also biased if we only consider the prevalence of variants in these 22 genes, aggregate or individually, although the relative effect will be smaller for genes with a higher prevalence of pathogenic variants. Comparisons of our cohort with that of Gröbner et al. do not suffer from this bias and similar results were obtained.

The purpose of our study was to estimate the prevalence of germline pathogenic variants in 22 cancer susceptibility genes, previously described by Zhang et al., in Swedish pediatric cancer patients and to obtain insights into the contribution of genetic predisposition to childhood cancer. It is highly likely that there are indeed germline mutations in other genes not analyzed in this study, as well as epigenetic alterations underlying the different pediatric cancers, and that the percentage of familial pediatric tumors is higher than observed here. Considering that we were able to identify a prevalence of germline mutations among pediatric cancer patients that is comparable to those described in recent broader screening studies^5,6^ in this limited analysis of 22 genes, suggests that these 22 genes harbor a substantial fraction of germline mutations in cancer susceptibility genes carried by pediatric cancer patients.

We found that the most commonly affected genes were *NF1*, *TP53*, the majority of which are seen in cancers associated with the predisposition syndromes neurofibromatosis and Li-Fraumeni syndrome, respectively. These cancers include CNS tumors such as optic glioma and astrocytoma resulting from *NF1* mutations, and osteosarcoma, soft-tissue sarcoma and ACT resulting from *TP53* mutations. We also found mutations in genes with no reported association to the diagnosis of the patient, such as *BRCA1* and *PTCH1* mutations in neuroblastoma, as well as *PMS2* mutations in leukemia. Incidental findings such as these are not uncommon when screening multiple cancer susceptibility genes and do not imply causation. Observed frequencies of these mutations are not inconsistent with those in the general population. For example, the frequency of *BRCA1* pathogenic variants in healthy non-Finnish European controls in gnomAD is 0.38% (81 of 21,384) compared to 0.25% in our cohort^23^.

We did not observe any significantly higher incidence of cancer among relatives of patients with germline mutations in cancer predisposition genes. This is in line with the findings reported by Zhang et al. While no significant association was found in either study, a numerical difference was found in our study when comparing cancer diagnoses among relatives of patients with and without germline mutations: 28% vs. 42%. This may suggest that a trend may emerge in investigations on a larger number of patients and/or broader genetic analyses including more variants.

In addition to identifying germline mutations in the tumor-bearing patients, a study by Kuhlen et al. highlighted the importance of assessing the presence of heterozygous mutations in the parents affecting the germline of the children, a procedure they termed ‘trio sequencing’^20,24^. This may help to identify mutations that could be candidates for familial surveillance with the aim of early detection and treatment. Implementation of surveillance has resulted in increased long-term survival of cancer patients from families with predisposition syndromes^25^. We currently have parent blood samples from a substantial number of patients presenting with germline mutations in our cohort, and trio sequencing studies are being planned together with larger-scale whole-genome sequencing approaches to examine genetic events that could have been overlooked in the highly focused analysis in the current study.

## Materials and methods

### Patients

The patients included in this study have been described previously^4^. In brief, the LCCG study enrolls pediatric cancer patients that are diagnosed and treated at the Skåne University Hospital in Sweden, including cancer survivors that are seen at the Late Effects Clinic. Patients are eligible for inclusion if diagnosed before the age of 18 years. The Swedish National Population Register was used to identify all relatives of patients up to the third-degree of relation. The Swedish Cancer Register was used to identify any cancer diagnoses of all relatives within the families of the patients up to the third-degree of relation.

### Sequencing and variant classification

Sequencing libraries were prepared from germline DNA extracted from 790 blood samples from the childhood cancer patients using the Fluidigm Juno technique. The assayed genes included the 21 autosomal dominant cancer predisposition genes for which pathogenic- or likely pathogenic variants were detected by Zhang et al.^5^, plus *ATM* (Table 1).

At least two libraries were prepared from all samples to maximize the sensitivity. Libraries were sequenced on an Illumina HiSeq 2500 system. The pathogenicity of the identified variants was determined according to ACMG-AMP (American College of Medical Genetics and Genomics—American College of Pathology) guidelines or ClinGen-approved gene-specific expert panel criteria, if available, in consultation with experts in clinical genetics and oncology at Lund University and the University of Amsterdam. Identified pathogenic variants were confirmed with Sanger sequencing and cross-referenced with patient clinical data and family history to identify associations with specific diagnoses as well as potential associations with increased familial cancer incidence. A detailed description of the sequencing and classification procedures is provided in Supplementary Methods, and the bioinformatic workflow is depicted in Supplementary Figure S1.

### Statistical analyses

Statistical comparisons were carried out using R statistical language (Version 3.3.1). The prevalence of diagnoses and of detected pathogenic variants in the sequenced genes were compared between cohorts using Fisher’s exact test and FDR-adjustments were applied to Fisher exact test *p*-values using p.adjust function from the stats (v3.1.1) R package with BH method^26^. For gene mutation prevalence comparisons, total cohort comparisons (including total cohort comparisons after removing TP53 mutations) were considered as one group of test for *p*-value adjustments, while all other individual gene test were considered a separate group of test. This also applies to mutation prevalence across different diagnoses, where total cohorts were considered one group and individual diagnoses analyses were considered a second group of tests. FDR-adjusted *p*-values (or *p*-values where applicable) < 0.05 were considered significant.

### Ethical approval

The study was approved by the Regional Ethics Review Board, Lund University, Sweden (no. 2008/233, 2010/231 and 2011/33). Access to the Population. Registry and Cancer Registry was approved for participants and parents. Written informed consent was received from patients and/or legal guardians prior to inclusion in this study and all research was performed in accordance with relevant guidelines/regulations.

## Supplementary Information

Supplementary Information 1.

Supplementary Information 2.

Supplementary Information 3.

## Abbreviations

LCCG: Lund childhood cancer genetics
SCCR: Swedish childhood cancer register
NHL: Non-Hodgkin’s lymphoma
ALL: Acute lymphoblastic leukemia
AML: Acute myeloid leukemia
ACT: Adrenocortical tumor
AF: Allele frequency
WGS: Whole genome sequencing

## References

[1] Saletta F, Dalla Pozza L, Byrne JA (2015). Genetic causes of cancer predisposition in children and adolescents. Transl Pediatr.

[2] Brodeur GM, Nichols KE, Plon SE, Schiffman JD, Malkin D (2017). Pediatric cancer predisposition and surveillance: an overview, and a tribute to Alfred G Knudson Jr. Clin Cancer Res.

[3] Magnusson S, Wiebe T, Kristoffersson U, Jernstrom H, Olsson H (2012). Increased incidence of childhood, prostate and breast cancers in relatives of childhood cancer patients. Fam Cancer.

[4] Stjernfelt KJ (2017). Predominance of girls with cancer in families with multiple childhood cancer cases. BMC Cancer.

[5] Zhang J (2015). Germline mutations in predisposition genes in pediatric cancer. N Engl J Med.

[6] Grobner SN (2018). The landscape of genomic alterations across childhood cancers. Nature.

[7] Stjernfelt KJ (2020). Increased cancer risk in families with pediatric cancer is associated with gender, age, diagnosis, and degree of relation to the child. Cancer Epidemiol Biomarkers Prev.

[8] Michalkiewicz E (2004). Clinical and outcome characteristics of children with adrenocortical tumors: a report from the international pediatric adrenocortical tumor registry. J Clin Oncol.

[9] Dominguez-Valentin M (2020). Cancer risks by gene, age, and gender in 6350 carriers of pathogenic mismatch repair variants: findings from the prospective Lynch syndrome database. Genet Med.

[10] Maniam P (2018). Pathogenicity and penetrance of germline SDHA variants in pheochromocytoma and paraganglioma (PPGL). J Endocr Soc.

[11] Ten Broeke SW (2018). Cancer risks for PMS2-associated Lynch syndrome. J Clin Oncol.

[12] Green RC (2013). ACMG recommendations for reporting of incidental findings in clinical exome and genome sequencing. Genet Med.

[13] Maris JM (2002). Evidence for a hereditary neuroblastoma predisposition locus at chromosome 16p12-13. Cancer Res.

[14] Origone P (2003). Homozygous inactivation of NF1 gene in a patient with familial NF1 and disseminated neuroblastoma. Am J Med Genet A.

[15] Crucis A (2015). Rhabdomyosarcomas in children with neurofibromatosis type I: a national historical cohort. Pediatr Blood Cancer.

[16] McPherson JR (2015). Whole-exome sequencing of breast cancer, malignant peripheral nerve sheath tumor and neurofibroma from a patient with neurofibromatosis type 1. Cancer Med.

[17] Correa H (2016). Li-Fraumeni syndrome. J Pediatr Genet.

[18] Campian J, Gutmann DH (2017). CNS tumors in neurofibromatosis. J Clin Oncol.

[19] Rahner N (2008). Compound heterozygosity for two MSH6 mutations in a patient with early onset colorectal cancer, vitiligo and systemic lupus erythematosus. Am J Med Genet A.

[20] Kuhlen M, Borkhardt A (2018). Trio sequencing in pediatric cancer and clinical implications. EMBO Mol Med.

[21] Qian M (2018). TP53 germline variations influence the predisposition and prognosis of B-cell acute lymphoblastic Leukemia in children. J Clin Oncol.

[22] Hameed M, Mandelker D (2018). Tumor syndromes predisposing to osteosarcoma. Adv Anat Pathol.

[23] Petridis C (2019). Frequency of pathogenic germline variants in BRCA1, BRCA2, PALB2, CHEK2 and TP53 in ductal carcinoma in situ diagnosed in women under the age of 50 years. Breast Cancer Res.

[24] Kuhlen M (2018). Family-based germline sequencing in children with cancer. Oncogene.

[25] Villani A (2016). Biochemical and imaging surveillance in germline TP53 mutation carriers with Li-Fraumeni syndrome: 11 year follow-up of a prospective observational study. Lancet Oncol.

[26] Team, R. C. Foundation for Statistical Computing. *A Language and Environment for Statistical Computing* (2016).

