# Supplementary material for: Prevalence of germline pathogenic variants in 22 cancer susceptibility genes in Swedish pediatric cancer patients
Source: Sci Rep. 2021 Mar 5;11:5307. doi: 10.1038/s41598-021-84502-4 (PMC7935871; doi:10.1038/s41598-021-84502-4)
Supplement: Supplementary file 3 — Supplementary Information 3. [file 41598_2021_84502_MOESM3_ESM.pdf]

Prevalence of germline pathogenic variants in 22 cancer susceptibility genes in Swedish pediatric cancer patients

Kristoffer von Stedingk[*]*, Karl-Johan Stjernfelt, Anders Kvist, Cecilia Wahlström, Ulf Kristoffersson, Marie Stenmark-Askmalm, Thomas Wiebe, Lars Hjorth, Jan Koster, Håkan Olsson*[#]*, Ingrid Øra*[#]

[*]Corresponding Author, [#]Shared Senior Authorship

Supplementary Information

Supplementary Figures

Supplementary Figure S1

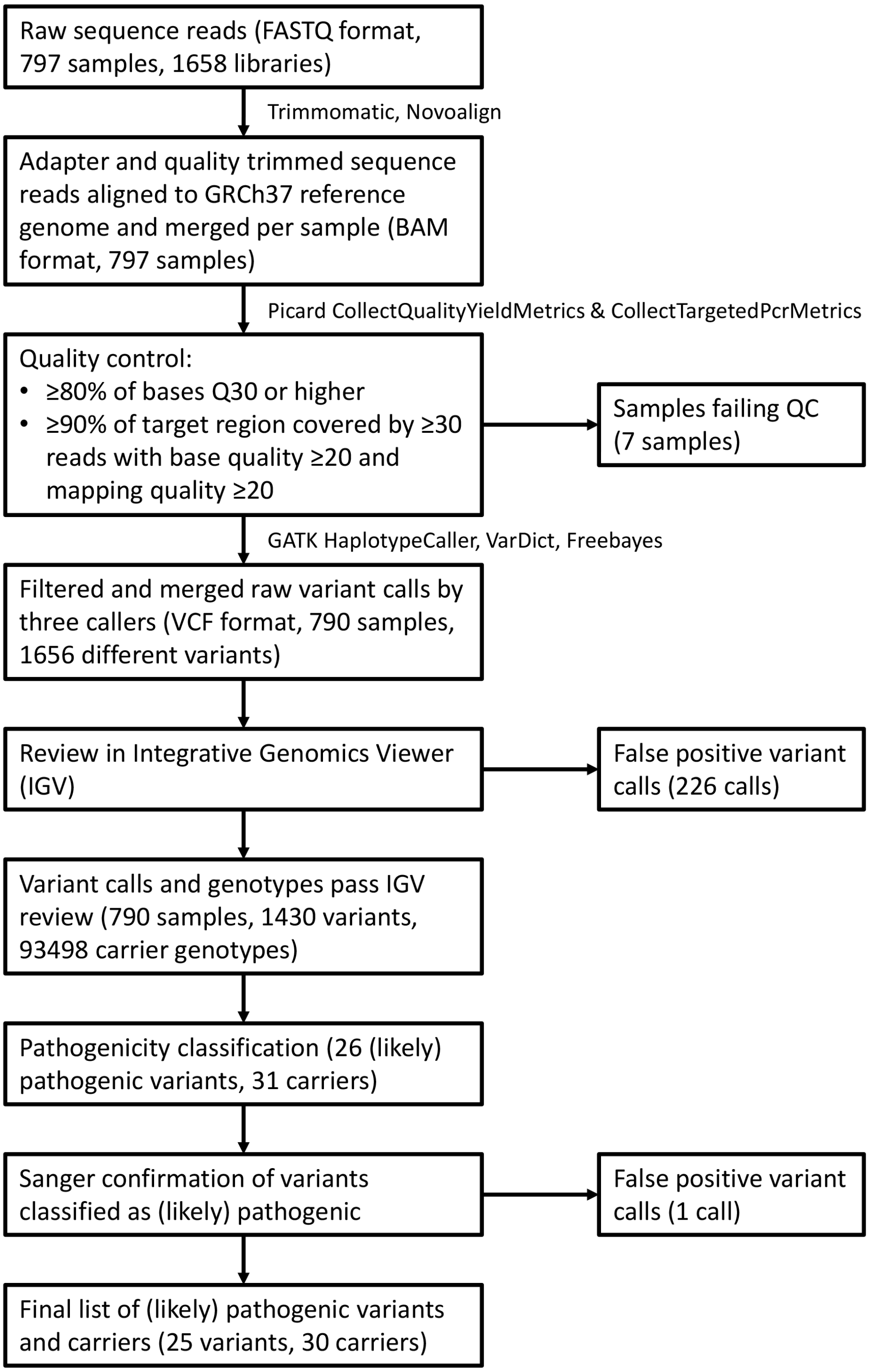

Supplementary Figure S1: Flowchart showing bioinformatic workflow for sequencing and variant identification/classification.

Supplementary Figure S2

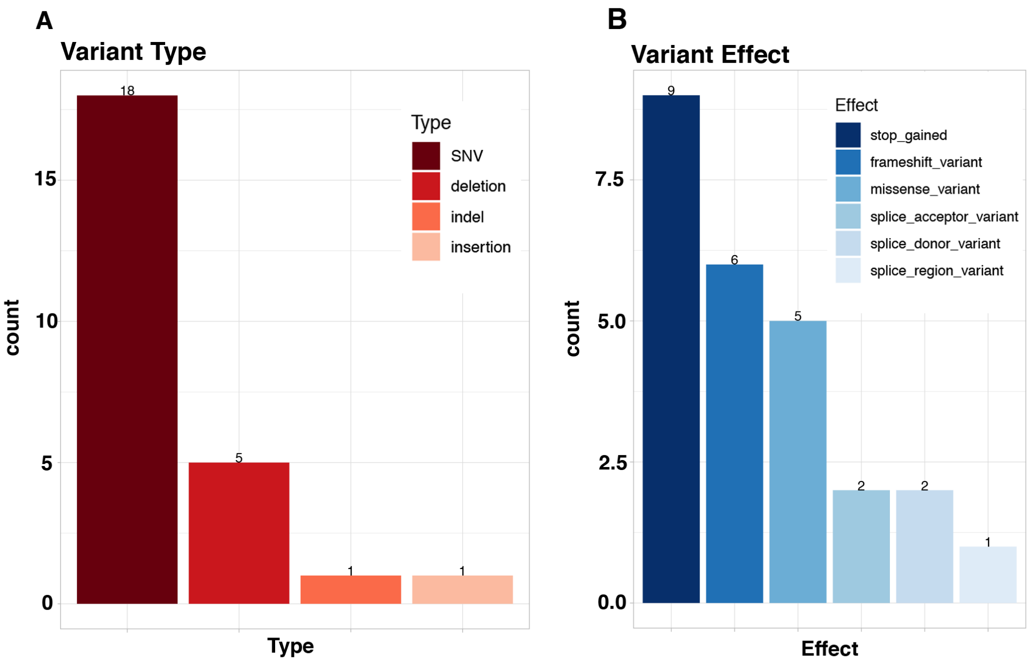

Supplementary Figure S2: Distribution of (A) variant type and (B) effect classification for the 25 variants determined to be pathogenic or likely pathogenic. Actual numbers are given above the bars.

Supplementary Methods

Target enrichment and sequencing

Fluidigm Juno target-enriched libraries were prepared for all 797 DNA samples. Since the final sequence yield of different libraries showed a degree of random variation not related to sample input DNA concentration, with some libraries having very low yield, at least two replicate libraries were prepared for each DNA sample (Supplementary Table "Library Info"). In total, 1706 sequencing libraries were prepared and sequenced. Two replicate libraries were sequenced for 773 of the DNA samples, four libraries for 23 of the samples, and 20 libraries for one of the samples. All samples passed our minimum base quality score requirement of 80% of bases at base quality 30 or higher. However, seven samples had less than 90% of the assay target region covered by 30 high-quality aligned reads. These seven samples were therefore excluded from further analyses. For the remaining samples, 94.6% of the target region was covered by 30 or more high-quality aligned reads, on average, and the mean sequence coverage was 1741 reads. Only 1.1% of the assay target region had no coverage, on average per sample (Supplementary Table "Sample Info")

Target-enrichment panel primer design

We designed a Fluidigm Juno targeted DNA sequencing assay (Fluidigm, South San Francisco, California, USA) for 22 genes previously found to carry pathogenic or likely pathogenic variants predisposing for cancer in a cohort of pediatric cancer patients (1). The assay was designed to target the coding region, 5′ untranslated region (5′ UTR) and 20 bp of intron adjacent to each exon/intron border of all complete coding transcripts from RefSeq and Gencode version 24 with transcript support level 1 (Supplementary Methods Tables "Target Transcripts" and "Target Summary"). The assay was performed using Fluidigm D3 software. Overlapping amplicons between 150 and 290 bp long were tiled across the target regions taking into account SNPs from dbSNP 137 with ≥1% frequency or flagged as clinically

associated. The forward and reverse primers included a universal 5′ tail sequence required for adding sample-specific index and adapter sequences for Illumina sequencing. The design was manually reviewed, and five amplicons with both primers designed in repeat regions were removed. Primer pairs were divided into multiplex pools based on melting temperature, and to avoid unintended complementarity between primers and primers and products. The final assay contained primer pairs for 809 amplicons divided into twenty primer pools (Supplementary Methods Table "Assay Amplicons"). The amplicons covered 97.97% of the intended target region and 99.66% of the coding sequence of the target gene transcripts (Supplementary Methods Table "Assay Design Efficiency").

DNA extraction, library preparation, target enrichment and sequencing

DNA was extracted from 797 samples using the QIAmp DNA Blood Maxi kit (Qiagen). Library preparation and target enrichment were performed using the Fluidigm Juno targeted DNA sequencing library preparation system with LP 192.24 integrated fluidic circuits (IFC), according to the manufacturer's protocol (Targeted Sequencing Preparation with Juno, Getting Started Guide, PN 101-0414 B1). Sample DNA concentrations were measured using a Nanodrop 8000 spectrophotometer (Thermo Fisher Scientific, Waltham, Massachusetts, USA). Samples with concentrations outside the specified input range (50- 200 ng/µl) were diluted or concentrated to a final concentration of 100 ng/µl. PCR master mix, sample-specific i7 index primers, and DNA from 192 different samples were combined into the 192 separate sample inlets of one IFC using a Biomek 4000 automated liquid handler (Beckman Coulter, Indianapolis, Indiana, USA). The twenty target-specific primer pools were loaded into the assay inlets and the IFC was loaded on the Fluidigm Juno instrument. The automated microfluidics system then amplified each DNA sample with each of the twenty primer pools, adding universal tail sequences and sample-specific i7 indexes. The resulting 192 individually indexed, target-enriched libraries were then harvested from the IFC as a single pool.

The IFC pool was cleaned up using SPRI beads and then amplified to add adapter sequences required for Illumina sequencing and an i5 index to identify the IFC pool. After a final clean-up, the IFC pool was measure using a Qubit (Qubit dsDNA HS Assay Kit, Invitrogen, Thermo Fisher Scientific) and an Agilent 2100 Bioanalyzer (High Sensitivity DNA Kit, Agilent, Santa Clara, California, USA) for quality control. The IFC pools were then pooled into larger pools for sequencing. These sequencing pools were again quality controlled using Qubit and the Agilent 2100 Bioanalyzer. With 384 available i7 indexes and four i5 indexes, up to 1,536 unique dual-indexed libraries can be pooled for sequencing. We created two sequencing pools, the first containing 960 libraries from five IFCs, two of which were negative controls (EB Buffer, Qiagen), and the second containing 768 libraries from four IFCs, 20 of which were negative controls.

The two library pools were sequenced on an Illumina HiSeq 2500 in 2 x 150 bp paired-end mode with dual 10 bp index reads and SBS V3 chemistry (Illumina, San Diego, California, USA). Each library pool was sequenced on two Illumina Rapid flow cells to provide sufficient sequence coverage. The concentrations of the sequencing pools were quantified using KAPA Library Quantification Kit Illumina platforms (KapaBiosystems, Wilmington, Massachusetts, USA) on a CFX96 Real-Time PCR System (Bio-Rad, Hercules, California, USA), and an aliquot was diluted to optimal concentration for loading on the sequencing flow cell. PhiX control library v3 was spiked in at a low concentration (~2%) for quality control (Illumina, FC-110-3001). At least two replicate libraries were prepared for each DNA sample.

Sequence preprocessing, alignment, and variant calling

See Supplemental figure S1 with bioinformatics processing workflow

Demultiplexing

Raw sequence data were demultiplexed according to the sample-specific i5 and i7 index sequences using ExtractIlluminaBarcodes and IlluminaBasecallsToFastq from Picard tools (version 1.128-4, http://broadinstitute.github.io/picard). Default parameters were used except MAX_MISMATCHES (which was set to 2), MIN_MISMATCH_DELTA (which was set to 2), ADAPTERS_TO_CHECK (set to null), APPLY_EAMSS_FILTER (set to false) and INCLUDE_NON_PF_READS (set to false).

Sequence read preprocessing

We trimmed adapter sequences from reads using Trimmomatic version 0.33 (2) with settings ILLUMINACLIP:<adapters.fasta>:3:12:7:1:true and MINLEN:30. Thereafter, low-quality sequences were trimmed with settings MAXINFO:30:0.25 and MINLEN:30. Remaining sequence reads from the PhiX control were identified and removed using bowtie2 alignment version 2.2.6 (3) using the PhiX reference genome NC_001422.1. Default parameters were used except -k (set to 1), --very-fast-local, --local--no-unal, --no-discordant and –dovetail.

Alignment

Sequence reads were aligned to the human reference genome using the base quality aware Novoalign version 3.02.13 (Novocraft Technologies Sdn Bhd, Petaling Jaya, Selangor, Malaysia, http://www.novocraft.com/products/novoalign/). We used the human reference genome GRCh37 with decoy sequences from the 1000 Genomes Project as reference (see ftp://ftp.1000genomes.ebi.ac.uk/vol1/ftp/technical/reference/phase2_reference_assembly_sequence/README_human_reference_20110707). To reduce allelic bias in the alignment, substitution variant alleles in dbSNP version 37 with an allele frequency of 1% or more in 1000 Genomes Project phase 1 genotype data were added to the reference genome as ambiguity codes. The Novoalign genome index was created with a k-mer length of 14 (-k 14)

and step size of 2 (-s 2). Novoalign was run in amplicons mode with a delta value of 2 (-amplicons <amplicon primer coordinates bed> 2). This means that sequences corresponding to the PCR primers are utilized for the alignment, but then soft-clipped from the ends of read-pairs mapping within 2 bp from the coordinates of the primer binding sites. Soft-clipping was enabled and a soft-clip bonus of 20 was added to the alignment score for alignments extending to the end of reads (--softclip 20). Other non-default parameters were -r R -Q2Off and -i +- 145-295 (the range of amplicon sizes in the assay is 150-290 bp). Read group information was added (AddOrReplaceReadGroups) and bam files merged per library (MergeSamFiles) using Picard version 2.8.2.

Sequence and alignment quality control

The yield (total reads and bases that pass the Illumina quality control "chastity" filter) and base qualities (percent bases that achieve a quality score of 30 or higher) of the demultiplexed, trimmed and filtered sequence reads were assessed using Picard CollectQualityYieldMetrics. Alignment quality control metrics were calculated using Picard TargetedPcrMetrics with MINIMUM_MAPPING_QUALITY set to 20, MINIMUM_BASE_QUALITY set to 20, and CLIP_OVERLAPPING_READS set to true. Evaluated quality metrics included PCT_PF_UQ_READS_ALIGNED, PCT_OFF_AMPLICON, MEAN_TARGET_COVERAGE, ZERO_CVG_TARGETS_PCT, and PCT_TARGET_BASES_30X (for definition of metrics, see http://broadinstitute.github.io/picard/picard-metric-definitions.htm). With these minimum mapping and base quality settings, the PCT_TARGET_BASES_30X can be regarded as a measure of the fraction of the target region that is "callable", meaning that the coverage of high-quality aligned reads is sufficient for a variant caller to detect genetic variants.

Samples were excluded from further analyses if less than 80% of bases had a quality score of 30 or higher, or if less than 90% of the target region was covered with 30 or more high quality aligned reads.

Variant calling

Discovery and genotyping of genetic variants was performed using three separate variant callers: HaplotypeCaller from GATK (version 3.7) (4), Freebayes (version 1.1.0-3-g961e5f3, Garrison et al. 2012, http://arxiv.org/abs/1207.3907), and VarDict (Java implementation version 1.5.0) (5).

- HaplotypeCaller default parameters were used apart from -useNewAFCalculator, -maxReadsInMemoryPerSample (set to 400,000), -maxReadsInRegionPerSample (set to 200,000) and --minReadsPerAlignmentStart (set to 30).
- Freebayes was run with default parameters apart from the following: --strict-vcf --pooled-discrete --pooled-continuous --no-partial-observations --no-population-priors --hwe-priors-off --binomial-obs-priors-off --allele-balance-priors-off --report-genotype-likelihood-max --genotype-qualities --ploidy 2 --use-best-n-alleles 6 --min-repeat-entropy 1 --min-alternate-fraction 0.1 --min-alternate-count 5 --min-base-quality 10.
- For VarDict the following parameters were set: -F 0x500 -I 120 -k 1 -z 1 -c 1 -S 2 -E 3 -g 4 -C -f 0.1 -r 5 -P 5 -q 25 -o 1.5 -O 0 -m 8 -Q 1 -a 10:0.95 -x 0. The VarDict output was piped through teststrandbias.R and var2vcf_valid.pl (non-default options: -a -E -P 0 -v 5 -f 0.1 -F 0.1).

Variant calling was performed using the merged per sample bam files containing data from all replicate libraries for each sample.

Variant call annotation and filtering

Variant calls were annotated using GATK VariantAnnotator adding the following annotations: Coverage (DP), QualByDepth (QD), RMSMappingQuality (MQ), MappingQualityRankSumTest (MQRankSum), BaseQualityRankSumTest (BaseQRankSum) and DepthPerAlleleBySample (AD). Descriptions of the annotations can be found in the GATK documentation (https://software.broadinstitute.org/gatk/documentation/tooldocs/3.8-0/). HaplotypeCaller and Freebayes, but not VarDict, also calculate a genotype quality (GQ) for each genotype call. Allele balance (AB) was calculated using a custom script as AD of the alternative allele divided by the total AD. Note that this differs from the allele balance calculated by GATK VariantAnnotator.

To filter out false calls from high-confidence variant calls we applied a set of hard filters that were optimized and validated using an independent data set obtained using a similar Fluidigm Juno panel and identical methods (see below). Variant calls that met any of the following criteria were removed: DP < 30, QUAL < 200 (HaplotypeCaller and Freebayes) or QUAL < 150 (VarDict), QD < 5.0, GQ < 50, AB < 0.2 for heterozygous calls, AB < 0.9 (FreebayesB and VarDict), or AB < 0.1 and AB < 0.9 (HaplotypeCaller) for homozygous calls. The following additional filters were applied to single nucleotide variants: MQ < 30, MQRankSum < -12.5, BaseQRankSum < -12.5 (HaplotypeCaller and Freebayes) or BaseQRankSum < -6.0 (VarDict).

The same genetic variant call can be represented in many different ways. In order to merge variant and genotype calls from different callers, variant calls were normalized using the variant tool vt (version 0.5772, (6)). Variant calls and genotype calls from the different callers were then merged using a custom Perl script. The script also divided vcf entries with multiple

ALT alleles into separate entries and normalized the representation of the separated alleles as needed.

Variant effect annotations

Ensembl Variant Effect Predictor (VEP, human release 94, version GRCh37, McLaren et al. 2016 (7)) was used to add annotations to aid evaluation of the potential pathogenicity of the detected variant alleles. Added annotations included Sequence Ontology consequence terms, and splice site consequence predictions using the plugins SpliceRegion and MaxEntScan (with options SWA and NCSS, Shamsani et al. 2018 (8)). Only RefSeq transcripts corresponding to the locus Reference Genomic (LRG, MacArthur et al. 2014 (9)) transcripts of the 22 target genes were used for annotation (Supplementary Methods Table "Annotation Transcripts"). Allele frequency annotations were taken from the Genome Aggregation Database (gnomAD, release 2.1, (10)) excluding samples from cancer cohorts. Evolutionary conservation scores (phastCons and phyloP), based on multiple alignments of 100 vertebrate species, were downloaded from the UCSC genome browser 2019-01-16 (11, 12). Potential deleterious effects of missense variants on protein function were predicted using Sift, Polyphen, and two *in silico* ensemble predictors: BayesDel (13) and Revel (14). For *TP53* missense variants, optimized Align-GVGD and BayesDel predictions were taken from Fortuno et al. (15). Summary annotations from ClinVar aggregate variation reports were extracted from the variant_summary.txt.gz file downloaded 2019-01-08 (ftp://ftp.ncbi.nlm.nih.gov/pub/clinvar/tab_delimited/, (16)). Clinvar, Human Gene Mutation Database (HGMD, (17)) and locus-specific databases were searched for information to aid classification (Supplementary Methods Table "Gene Disease LSDBs").

Variant pathogenicity classification

We used the American College of Medical Genetics and Genomics (ACMG) and American College of Pathology (AMP) framework for variant classification to evaluate the potential clinical significance of the detected variant alleles (18), with a few modifications specified below, except for the genes that had ClinGen-approved gene-specific expert panel criteria for classification at the time of writing (*CDH1, MSH2, MSH6, PMS2, BRCA1 and BRCA2*). For these genes the criteria developed by the expert panels were used (*CDH1*: Lee et al. 2018 (19), *MSH2, MSH6, PMS2*: criteria developed by the InSiGHT Variant Interpretation Committee, https://www.insight-group.org/content/uploads/2018/08/2018-06_InSiGHT_VIC_v2.4.pdf, *BRCA1, BRCA2*: criteria developed by the ENIGMA consortium, https://enigmaconsortium.org/wp-content/uploads/2018/10/ENIGMA_Rules_2017-06-29-v2.5.1.pdf). For variants already classified by a ClinGen-approved variant curation expert panel, the expert panel classifications were used.

We applied the ACMG/AMP PVS1 criterion for loss of function variants at different strength levels depending on the type and features of the specific variant, according to the ClinGen Sequence Variant Interpretation Group recommendations (20). The PP5 and BP6, BP1 (missense variant in a gene for which primarily truncating variants are known to cause disease), and PM3 (for recessive disorders) were not used. The PP3 and BP4 criteria were applied to TP53 missense variants according to suggestions by Fortuno et al. (15) based on optimized alignGVGD combined with BayesDel predictions. Variants were evaluated for their potential effect on splicing using MaxEntScan and thresholds from the ENIGMA BRCA1/2 classification guidelines (8).

The vast majority of pathogenic variants in genes associated with hereditary cancers have allele frequencies well below 0.01% in large outbred general population databases (21). The very few pathogenic variants with allele frequencies above 0.01% in the general population are likely to be well known, already characterized in the literature, and represented in databases of pathogenic variants such as ClinVar (21). We therefore applied the ACMG/AMP BS1 criterion with an allele frequency cutoff of 0.01%, except for variants already reported as (likely) pathogenic by any submitter to ClinVar. To account for sampling variance, we used a so-called "filtering allele frequency" threshold, the maximum true population allele frequency that is consistent with a particular allele count observed in a population based on the Poisson 95% confidence interval (22). Filtering allele frequencies from populations known to have gone through a bottleneck (Ashkenazi Jewish and Finnish) were not used. We applied the BA1 criterion to variants with a filtering allele frequency greater than 1%. The BS2 criterion was applied as supporting evidence (BS2_P) in combination with BS1 if homozygous individuals were reported in gnomAD or ExAc populations.

Confirmation of variants classified as (likely) pathogenic

The supporting alignment data for all variants classified as pathogenic or likely pathogenic were visually reviewed in Integrative Genomics Viewer (IGV) to help identify potential false positives and characterize complex variants (23, 24). All variants classified as pathogenic and likely pathogenic that passed the review in IGV were Sanger sequenced to confirm true variant calls.

Validation of methods

We have validated the Fluidigm Juno method using a larger assay covering 31 genes and 1349 amplicons in 20 primer pools. That assay included 10 of the 22 genes and 56% of the targeted bases of the assay used in this study. The methods used for assay design, library preparation, target enrichment, sequencing, and bioinformatic analyses were identical to those used in this study and described above with one exception: we removed amplicons with both primers in repeat regions when designing the panel used in this study. This reduced the off-target capture in this study compared to the validation study. Amplicon lengths were similar with an average of 219 bp in the validation design and 222 bp in this study design.

We used 95 DNA samples extracted from blood as positive controls. These DNA samples had previously been screened for genetic variants using a clinically validated hybrid selection assay (Agilent SureSelect) and were selected to represent a broad variety of sequence variants. The overlap between the target regions of the clinical hybrid selection assay and the Fluidigm Juno validation assay included the coding region and 10 bp adjacent introns of 21 genes (78,643 bp), and the positive controls together contained 314 different genetic variants in that region. These variants included both common polymorphisms and rare variants. The longest deletion was 21 bp and the longest insertion was 11 bp combined with a deletion of 1 bp. Most control variants were also Sanger sequenced for confirmation (96% of the indels and 89% of the SNVs).

The sequence yield and coverage were similar for the positive control libraries prepared with the validation assay and the libraries prepared with the assay used in this study. On average,

95.1% of the bases targeted by the validation panel were covered with 30 or more high-quality aligned reads, compared to 94.6% in this study.

The sensitivity of the validation assay to detect the positive control variants was 96.7%, calculated as the mean sensitivity across variants ($S_{mean(var)}$, see below). The number of false positive variants (FP) detected was 41 (11.5%). All the false positives were identified as such when visually reviewed in IGV. The fraction of false positives identified when variants detected in this study were reviewed in IGV was similar (13.4%) to the false positive rate estimated for the validation assay.

The sensitivity per variant (S_{var}) was calculated as:

$$S_{var} = \frac{TP_{var}}{(TP_{var} + FN_{var})}$$

where TP_{var} is the number of libraries with true positive calls, and FN_{var} is the number of libraries with false negative calls for the variant.

The mean sensitivity across variants ($S_{mean(var)}$) was calculated as

$$S_{mean(var)} = \frac{S_{var}}{N_{var}}$$

where N_{var} is the number of distinct variants. This mean gives equal weight to each distinct variant, rare or common, and is therefore a good measure of our ability to detect the complete spectrum of variants.

Supplemental Methods References

1. Zhang J, et al. Germline Mutations in Predisposition Genes in Pediatric Cancer. *N Engl J Med.* 2015;373(24):2336-46.
2. Bolger AM, Lohse M, and Usadel B. Trimmomatic: a flexible trimmer for Illumina sequence data. *Bioinformatics.* 2014;30(15):2114-20.
3. Langmead B, and Salzberg SL. Fast gapped-read alignment with Bowtie 2. *Nat Methods.* 2012;9(4):357-9.
4. McKenna A, et al. The Genome Analysis Toolkit: a MapReduce framework for analyzing next-generation DNA sequencing data. *Genome Res.* 2010;20(9):1297-303.
5. Lai Z, et al. VarDict: a novel and versatile variant caller for next-generation sequencing in cancer research. *Nucleic Acids Res.* 2016;44(11):e108.
6. Tan A, Abecasis GR, and Kang HM. Unified representation of genetic variants. *Bioinformatics.* 2015;31(13):2202-4.
7. McLaren W, et al. The Ensembl Variant Effect Predictor. *Genome Biol.* 2016;17(1):122.
8. Shamsani J, et al. A plugin for the Ensembl Variant Effect Predictor that uses MaxEntScan to predict variant spliceogenicity. *Bioinformatics.* 2018.
9. MacArthur JA, et al. Locus Reference Genomic: reference sequences for the reporting of clinically relevant sequence variants. *Nucleic Acids Res.* 2014;42(Database issue):D873-8.
10. Lek M, et al. Analysis of protein-coding genetic variation in 60,706 humans. *Nature.* 2016;536(7616):285-91.
11. Pollard KS, Hubisz MJ, Rosenbloom KR, and Siepel A. Detection of nonneutral substitution rates on mammalian phylogenies. *Genome Res.* 2010;20(1):110-21.
12. Siepel A, et al. Evolutionarily conserved elements in vertebrate, insect, worm, and yeast genomes. *Genome Res.* 2005;15(8):1034-50.
13. Feng BJ. PERCH: A Unified Framework for Disease Gene Prioritization. *Hum Mutat.* 2017;38(3):243-51.
14. Ioannidis NM, et al. REVEL: An Ensemble Method for Predicting the Pathogenicity of Rare Missense Variants. *Am J Hum Genet.* 2016;99(4):877-85.
15. Fortuno C, et al. Improved, ACMG-compliant, in silico prediction of pathogenicity for missense substitutions encoded by TP53 variants. *Hum Mutat.* 2018;39(8):1061-9.
16. Landrum MJ, et al. ClinVar: improving access to variant interpretations and supporting evidence. *Nucleic Acids Res.* 2018;46(D1):D1062-D7.
17. Stenson PD, et al. Human Gene Mutation Database (HGMD): 2003 update. *Hum Mutat.* 2003;21(6):577-81.
18. Richards S, et al. Standards and guidelines for the interpretation of sequence variants: a joint consensus recommendation of the American College of Medical Genetics and Genomics and the Association for Molecular Pathology. *Genet Med.* 2015;17(5):405-24.
19. Lee K, et al. Specifications of the ACMG/AMP variant curation guidelines for the analysis of germline CDH1 sequence variants. *Hum Mutat.* 2018;39(11):1553-68.
20. Abou Tayoun AN, et al. Recommendations for interpreting the loss of function PVS1 ACMG/AMP variant criterion. *Hum Mutat.* 2018;39(11):1517-24.

21. Kobayashi Y, Yang S, Nykamp K, Garcia J, Lincoln SE, and Topper SE. Pathogenic variant burden in the ExAC database: an empirical approach to evaluating population data for clinical variant interpretation. *Genome Med.* 2017;9(1):13.
22. Whiffin N, et al. Using high-resolution variant frequencies to empower clinical genome interpretation. *Genet Med.* 2017;19(10):1151-8.
23. Robinson JT, et al. Integrative genomics viewer. *Nat Biotechnol.* 2011;29(1):24-6.
24. Thorvaldsdottir H, Robinson JT, and Mesirov JP. Integrative Genomics Viewer (IGV): high-performance genomics data visualization and exploration. *Brief Bioinform.* 2013;14(2):178-92.
